# Politics and Biliousness

**Published:** 1884-05

**Authors:** 


### POLITICS AND BILIOUSNESS.

*Le Figaro* publishes a most humorous article entitled "M. Challemel-Lacour's Liver," demonstrating that the destinies of the nation are at the mercy of a bilious man who signs treaties or threatens war according to the state of his liver at the moment he is discussing affairs with the representative of a foreign power.

